# Development and comprehensive validation of a predictive prognosis model for very early HCC recurrence within one year after curative resection: a multicenter cohort study

**DOI:** 10.1097/JS9.0000000000001467

**Published:** 2024-04-15

**Authors:** Lei Liu, Shangdong Qin, Kongying Lin, Qingguo Xu, Yuan Yang, Jinzhen Cai, Yongyi Zeng, Shengxian Yuan, Bangde Xiang, Wan Yee Lau, Weiping Zhou

**Affiliations:** a The Third Department of Hepatic Surgery, Eastern Hepatobiliary Surgery Hospital; b Hepatobiliary Surgery Department, Guangxi Medical University Cancer Hospital, Nanning; c Department of Hepatopancreatobiliary Surgery, Mengchao Hepatobiliary Hospital of Fujian Medical University, Fuzhou; d Organ Transplantation Center, The Institute of Transplantation Science, The Affiliated Hospital of Qingdao University; e Faculty of Medicine, The Chinese University of Hong Kong, Shatin, New Territories, Hong Kong SAR; f Key Laboratory of Signaling Regulation and Targeting Therapy of Liver Cancer (SMMU), Ministry of Education; g Shanghai Key Laboratory of Hepatobiliary Tumor Biology (EHBH), Shanghai, People’s Republic of China

**Keywords:** early recurrence, hepatocellular carcinoma, liver resection, prognostic predictive score

## Abstract

**Background:**

The high incidence of early recurrence after liver resection (LR) for hepatocellular carcinoma (HCC) is the main obstacle in achieving good long-term survival outcomes. The aim of the present study is to develop a prognostic model in predicting the risk of very early (1-year) recurrence.

**Material and methods:**

Consecutive patients who underwent LR for HCC with curative intent at multicenters in China were enrolled in this study. The VERM-pre (the Preoperative Very Early Recurrence Model of HCC) with good performance was derived and validated by internal and external cohorts retrospectively and by another two-center cohort prospectively.

**Results:**

Seven thousand four hundred one patients were enrolled and divided randomly into three cohorts. Eight variables (tumor diameter, tumor number, macrovascular invasion, satellite nodule, alpha-fetoprotein, level of HBV-DNA, γ-GT, and prothrombin time) were identified as independent risk factors for recurrence-free survival on univariate and multivariate analyses. The VERM-pre model was developed which showed a high capacity of discrimination (C-index: 0.722; AUROC at 1-year: 0.722)) and was validated comprehensively by the internal, external, and prospective cohorts, retrospectively. Calibration plots showed satisfactory fitting of probability of early HCC recurrence in the cohorts. Three risk strata were derived to have significantly different recurrence-free survival rates (low-risk: 80.4–85.4%; intermediate-risk: 59.7–64.8%; high-risk: 32.6–42.6%). In the prospective validation cohort, the swimming plot illustrated consistent outcomes with the beginning predictive score.

**Conclusion:**

The VERM-pre model accurately predicted the 1-year recurrence rates of HCC after LR with curative intent. The model was retrospectively and prospectively validated and then developed as the online tool.

## Introduction

Highlights

- The VERM-pre model accurately predicted the 1-year recurrence rates of hepatocellular carcinoma after liver resection with curative intent.
- The VERM-pre model was well-validated retrospectively and prospectively.
- The online tool of VERM-pre was available on the website: https://dr-ray.shinyapps.io/VERM-pre/.

Hepatocellular carcinoma (HCC) is a major cause of cancer-related death in the world and nearly half of new cases each year occurs in China^1,2^. Currently liver resection (LR) is still a commonly recommended treatment option for very early and early stages of HCC based on the BCLC staging^2,3^ and the American Joint Committee on Cancer (AJCC) Tumor-Node-Metastasis (TNM) systems^4,5^. However, postoperative recurrence of HCC after LR is still high^6^. Previous studies have confirmed tumor recurrence within 1-year after LR as an independent risk factor for poor overall survival (OS)^7–10^ probably because postoperative early recurrence suggests a more aggressive tumor biology. Thus, a high-quality prognostic predictive model should not only concern tumor burden (tumor number and tumor diameter) as what BCLC staging, AJCC TNM, and Chinese Liver Cancer (CNLC)^5,11,12^ classifications have done, but also incorporate tumor biology and liver underling disease parameters. Shim *et al*.^13^ suggests a Korean model, which included five parameters (tumor volume, vascular invasion, platelet, serum albumin, and sex) to predict 2-year recurrence-free survival (RFS) for HCC after LR using a nomogram. Another postoperative nomogram model named the Singapore Liver Cancer Recurrence (SLICER) Score has been developed for predicting the 3-year and 5-year RFS based on a single center study^14^. The Early Recurrence After Surgery for Liver tumor (ERASL) model is a linear model formed by an equation which incorporates five parameters and validated by three cohorts retrospectively^15^. In the past decades, many prognostic predictive models have been established, although few have been commonly used in clinical practice. The debatable reproducibility and retrospective validation outcomes limit clinical application. Moreover, some dichotomous models are considered as inferior to the comparable continuous risk scores^16,17^. Some researchers suggest that the model quality could be improved when the nonlinear prognostic factors and missing values are better addressed^18^. Given the overall consideration, in the present study we aimed to develop a linear mathematic preoperative prognostic predictive model, which incorporated the comprehensive and commonly available preoperative parameters and established by employing a large cohort of HCC patients after LR with curative intent from national-wide centers. The model was compared to the above mentioned models and staging scores and validated by internal-cohorts and external cohorts retrospectively, and also validated by a prospective observational study. The model was further developed as an online calculator to facilitate use to provide a more accurate prediction after LR with curative intent for HCC patients.

## Material and methods

### Statement of ethics

This study was approved by the Ethics Committee of Eastern Hepatobiliary Surgery Hospital (NO. EHBHKY 2023-K027- P001). Informed consents were obtained from all patients for their data to be used in clinical researches. This study was registered in the Chinese Clinical Trial Registry from http://www.chictr.org.cn (ChiCTR2300077747) and conducted in line with the Strengthening the reporting of cohort, cross-sectional and case-control studies in surgery (STROCSS) 2021 criteria^19^.

### Patients

Patients with HCC who underwent LR with curative intent at four centers in China (the Eastern Hepatobiliary Surgery Hospital, Guangxi Medical University Cancer Hospital, Mengchao Hepatobiliary Hospital of Fujian Medical University, The Affiliated Hospital of Qingdao University) from Jan 2002 to Jul 2020 were retrospectively studied. The inclusion criteria were patients with: 1) no anticancer treatment preoperatively; 2) no other known malignant tumors; 3) complete data; 4) Child-Pugh A or B7 (score ≤7) liver function and sufficient remnant liver volume as calculated by 3-dimenstional reconstruction of liver; 5) 18 ≤age <85 years; 6) no extrahepatic metastasis; 7) R0 LR. Patients who died within 30-day postoperatively were excluded.

Informed consent was obtained from all patients for the operation and for their data to be used for clinical research.

### Definition

Curative LR was defined as complete removal of all macroscopic tumors with negative surgical margins on microscopy^20,21^. Macrovascular invasion was determined by intravenous contrast enhanced CT or MRI. Satellite nodules were determined as small tumors separated by an interval of normal liver parenchyma within 2 cm on intravenous contrast enhanced CT or MRI^22–24^. Tumor capsule was assessed by radiology, and a complete capsule was defined as a smooth, sharply outlined area of tumor margin on portal venous phase images^25^. Very early recurrence was defined as postoperative recurrence within 1-year which was relative to the common early recurrence concept (2 years).

### Data collection and follow-up

Parameters collected included data on patient-related factors and preoperative examination results. Patients’ age, sex, and etiology of liver disease were reviewed. Preoperative investigations included blood tests (alpha-fetoprotein (AFP) level, prothrombin time (PT), platelet count, total bilirubin (TBil), γ-glutamyl transpeptidase (γ-GT), HBV-DNA and albumin levels); radiology-related data were obtained by MRI or CT (tumor size, tumor number, satellite nodule, tumor capsule, and macrovascular invasion).

Regarding antiviral treatment, if the patient has been previously undergoing antiviral therapy, the established treatment plan will be continued. If the patient has not undergone any antiviral treatment, our center typically administers antiviral therapy using entecavir or tenofovir. After hospital discharge, patients were followed-up for AFP levels, upper abdominal enhanced MRI or CT and chest CT once every 3 months in the first year, then once every 6 months subsequently. RFS was defined as the interval between the time of operation to the time of recurrence.

### The prospective observational validation study

For further validation of the models, a prospective observational study was conducted from January 2021. We recruited 297 patients who undertook LR for HCC under the similar inclusion and exclusion criteria in the Eastern Hepatobiliary Surgery Hospital (122 cases) and Mengchao Hepatobiliary Hospital of Fujian Medical University (175 cases). Each eligible patient was assessed by the present model and given the final prognostic predictive score before surgery. After hospital discharge, follow-up strategy was the same as described above. Informed consent was also obtained from all these patients.

### Statistical analysis

All statistical analyses were performed by R version 4.1.1 (R Foundation for Statistical Computing). Categorical variables were presented as number and percentages and compared by the *χ*^2^ test or the Fisher’s exact test. Continuous data were expressed as mean±SD or median with interquartile range (IQR) and compared using the Student’s *t*-test or the Mann–Whitney *U* test. The model in predicting early recurrence using preoperatively data was constructed based on the cut-off timing of 1-year. In the present study, variables were selected based on clinical practice and well-established factors associated with prognosis. For handling missing data, two methods were used: 1. If there was a large amount of missing data, it was excluded; 2. For less missing data, it was imputed using the mean or median. Univariate and multivariate cox regressive tests were used to identify independent risk factors. The multivariate cox model was constructed by stepwise backward selection of variables of variable significant at the 5% level, and confirmed by the minimum Akaike information criterion (AIC) value. The Schoenfeld residual test was used to verify the assumption of proportional hazards in the Cox analysis. Plots of scaled Schoenfeld residuals against time were examined for each variable in the model. Hazard ratios (HR) were computed by model β-estimates. The risk score function for prediction of very early recurrence was calculated based on the β-estimates. The best cut-off values of the model were induced via the X-tile software and patients were stratified into three risk groups (low, intermediate, and high). Survival curves of the three groups for the training, internal, external, and prospective validation cohorts were calculated and plotted by the Kaplan–Meier test. Capacity of the model discrimination was tested by the Harrell’s C-index and the area under the receiver operating characteristic (AUROC)^15,26^. Time-dependent receiver operating characteristic of the model was also plotted to illustrate variations of discrimination with time passing. The estimation of clinical utility and net benefit was conducted using DCA (decision curve analysis), demonstrating its practical value^27^. The model was compared with the ERSAL-pre, ERSAL-post, AJCC TNM, Barcelona Clinic Liver Cancer Staging (BCLC), Chinese Liver Cancer (CNLC) classification, Singapore Liver Cancer Recurrence (SLICER), and Korean model^2–5,12–15,28,29^. Calibration plots were generated to calibrate the present models. They were also applied to the internal and external validation cohorts. The swimming plot was depicted to illustrate the follow-up outcomes of the patients in the prospective study. To make the model user-friendly, the mathematical function was improved to develop an online calculator using the shinyPredict package.

## Results

### Patients

Of 7832 patients who underwent LR with curative intent during the study period, 140 patients were excluded because of preoperative treatments including TACE, sorafenib or Lenvatinib, 155 patients with incomplete data or were lost to follow-up, 30 patients had extrahepatic metastasis, 46 patients died within 30 days of operation and 60 patients with tumor rupture or positive resection margins. Eventually, 5534 patients (the Shanghai cohort) were equally divided randomly into two groups to form the training and internal validation cohorts. The external validation cohort consisted of 1867 patients from three other centers. In the prospective validation cohort, 297 eligible patients were recruited from January, 2021 to December, 2021. The flow chart of the above patients was shown in Supplement Figure 1 (Supplemental Digital Content 1 http://links.lww.com/JS9/C391). The baseline characteristics are summarized in Table 1. The number of patients who had recurrence within 1-year postoperatively in the training cohort was almost similar to the internal validation cohort (774 vs 771, 28 vs 27.9%), but was less than the external validation cohort (782, 41.9%), and there were 103 (35%) patients with 1-year recurrence in the prospective cohort.

**Table 1:** Baseline characteristics.

Variables	Training cohort	Internal validation cohort	External validation cohort	Prospective validation cohort
Total	*n*=2767	*n*=2767	*n*=1867	*n*=297
Age, years, median (IQR)				
	52 (44–59)	52 (44–60)	51 (44–60)	54 (47–65)
Sex, *n* (%)				
Female	383 (13.8)	376 (13.6)	256 (13.7)	67 (22.6)
Male	2384 (86.2)	2391 (86.4)	1611 (86.3)	230 (77.4)
Etiology, *n* (%)				
Hepatitis C	48 (1.7)	38 (1.4)	39 (2.1)	
Hepatitis B	2404 (86.9)	2414 (87.2)	1512 (81.0)	
Other	315 (11.4)	314 (11.4)	316 (16.9)	
Blood tests				
Platelet, ×10^9^, median (IQR)				
	154 (116–200)	158 (118–202.5)	194 (147–250.9)	165 (125–214.5)
Total bilirubin, g/l, median (IQR)				
	13.4 (10.4–17.3)	13.5 (10.4–17.3)	12.7 (9.5–16.9)	14 (10.7–18.1)
Albumin, g/l, median (IQR)				
	41.8 (39.3–44.1)	41.9 (39.5–44.3)	40.4 (37.1–43.5)	40 (37–43)
γ-glutamyl transpeptidase, U/l, median (IQR)				
	63 (35–116)	63 (37–117.5)	62 (35–115.9)	57 (31–106)
LN(γ-GT), median (IQR)				
	4.1 (3.6–4.8)	4.1 (3.6–4.8)	4.1 (3.6–4.8)	4.0 (3.4–4.7)
AFP, ng/ml, median (IQR)				
	85 (6.8–1210)	88 (6.3–1210)	183.5 (8.6–1400.7)	137.1 (13.1– 760.8)
LN(AFP), median (IQR)				
	4.4 (1.9–7.1)	4.5 (1.8–7.1)	5.2 (2.1–7.2)	4.9 (2.6–6.6)
Prothrombin time, s, median (IQR)				
	11.9 (11.3–12.6)	11.9 (11.3–12.5)	12.1 (11.4–12.8)	12.7 (12–13.5)
HBV-DNA, median (IQR)				
	1000 (500–43 400)	1000 (500–42 400)	1180 (1000–10 000)	1170 (50– 48 300)
Lg (HBV-DNA), median (IQR)				
	3 (2.7–4.6)	3 (2.7–4.6)	3.1 (3–5)	3.1 (1.7–4.7)
Tumor characteristics via radiological examination				
Tumor number, *n* (%)				
1	2229 (80.6)	2209 (79.8)	1446 (77.5)	166 (55.9)
2	343 (12.4)	330 (11.9)	314 (16.8)	104 (35.0)
3	87 (3.1)	117 (4.2)	47 (2.5)	21 (7.1)
4	86 (3.1)	85 (3.1)	23 (1.2)	5 (1.7)
5	22 (0.8)	26 (0.9)	37 (2)	1 (0.3)
Tumor size, cm, median (IQR)				
	5.1 (3.3–8.2)	5.2 (3.4–8.4)	6 (4–10)	5 (3.1–8.3)
Macrovascular invasion, *n* (%)				
Without	2438 (88.1)	2440 (88.2)	1436 (76.9)	–
With	329 (11.9)	327 (11.8)	431 (23.1)	–
Satellite nodule, *n* (%)				
Without	1461 (52.8)	1474 (53.3)	1592 (85.3)	258 (86.9)
With	1306 (47.2)	1293 (46.7)	275 (14.7)	39 (13.1)
Tumor capsule, *n* (%)				
Complete capsule	560 (20.2)	558 (20.2)	407 (21.8)	–
Partial capsule	1654 (59.8)	1636 (59.1)	1179 (63.1)	–
Without capsule	553 (20)	573 (20.7)	281 (15.1)	–
Surgical parameters				
Major hepatectomy, *n* (%)				
No	2202 (79.6)	2200 (79.5)	1492 (79.9)	–
Yes	565 (20.4)	567 (20.5)	375 (20.1)	–
Intraoperative blood bleeding, ml, median (IQR)				
	200 (100–300)	200 (100–300)	200 (100–300)	–
Intraoperative blood transfusion, *n* (%)				
No	2484 (89.8)	2495 (90.2)	1652 (88.5)	–
Yes	283 (10.2)	272 (9.8)	215 (11.5)	–
Histological parameters				
Tumor differentiation, *n* (%)				
1	6 (0.2)	10 (0.4)	64 (3.4)	–
2	302 (10.9)	320 (11.6)	458 (24.5)	–
3	2342 (84.6)	2322 (83.9)	1266 (67.8)	–
4	117 (4.2)	115 (4.2)	79 (4.2)	–
MVI, *n* (%)				
Without	1677 (60.6)	1662 (60.1)	1018 (54.5)	–
With	1090 (39.4)	1105 (39.9)	849 (45.5)	–
Surgical margins, cm, mean (SD)				
	0.4 (0.6)	0.4 (0.6)	0.6 (0.7)	–
Hepatic cirrhosis, *n* (%)				
Without	865 (31.3)	903 (32.6)	904 (48.4)	–
With	1902 (68.7)	1864 (67.4)	963 (51.6)	–
Recurrence, *n* (%)				
No	1610 (58.2)	1551 (56.1)	617 (33)	173 (58.2)
Yes	1157 (41.8)	1216 (43.9)	1250 (67)	124 (41.8)
Recurrence within 1-year, *n* (%)				
No	1993 (72)	1996 (72.1)	1085 (58.1)	187 (63)
Yes	774 (28)	771 (27.9)	782 (41.9)	110 (37)
Recurrence-free survival, months, median (IQR)				
	17.7 (5.6–42.9)	16.7 (5.6–40.5)	15 (4.5–35.6)	14 (9–17)
Postrecurrence survival, months, median (IQR)				
	21.4 (6.2–45.1)	22.3 (6.2–44.8)	15 (4–35)	–
Overall survival, months, median (IQR)				
	30.7 (12.5–50.5)	31.5 (12.3–51)	no data	–

AFP, alpha-fetoprotein; HBV-DNA, hepatitis B virus deoxyribonucleic acid; IQR, interquartile range; MVI, microvascular invasion.

Continuous data were expressed as mean±SD or median with interquartile range; Categorical data were expressed as number (percentage).

### Independent risk factors associated with very early HCC recurrence

Sixteen preoperative risk factors in the present study were tested statistically. The outcomes of univariate analysis are displayed in Supplement Table 1 (Supplemental Digital Content 2 http://links.lww.com/JS9/C401). Nine preoperative variables including macrovascular invasion, tumor number, tumor diameter, satellite nodule, tumor capsule, γ-GT, AFP, PT, and level of HBV-DNA were associated with very early HCC recurrence on univariate analysis based on *P*<0.1. On multivariate analysis, tumor capsule was excluded because its *P*>0.05. All the other parameters of histological tests and surgery were not significantly relevant. The results are summarized in Table 2.

**Table 2:** Multivariable analysis of prognostic parameters for the model in the training cohort.

	VERM-pre			
Parameters	Hazard ratio (95% CI)	Coefficient (95% CI)	*P*	AIC
Macrovascular invasion				
No	Ref	Ref		11442.7
Yes	2.181 (1.814–2.621)	0.780 (0.596–0.964)	<0.001	
Tumor number	1.145 (1.056–1.242)	0.136 (0.054–0.217)	0.001	
Tumor diameter	1.064 (1.045–1.084)	0.062 (0.044–0.081)	<0.001	
Satellite nodule				
No	Ref	Ref		
Yes	1.214 (1.034–1.426)	0.194 (0.033–0.355)	0.018	
Ln (γ-GT)	1.175 (1.071–1.289)	0.161 (0.069–0.254)	0.001	
Ln (AFP)	1.106 (1.070–1.143)	0.101 (0.068–0.134)	<0.001	
PT	1.125 (1.049–1.206)	0.118 (0.048–0.187)	0.001	
Lg (HBV-DNA)	1.051 (1.003–1.101)	0.050 (0.003–0.096)	0.038	

AFP, alpha-fetoprotein; AIC, Akaike information criterion; HBV-DNA, hepatitis B virus deoxyribonucleic acid; PT, prothrombin time; γ-GT, γ-glutamyl transpeptidase.

VERM-pre, the preoperative Very Early Recurrence Model, VERM-pre = 0.780* macrovascular invasion (0: no;1: yes) +0.136* Tumor number +0.062* Tumor diameter in cm+0.194* Satellite nodule (0: no; 1: yes) +0.161* Ln (γ-GT in U/L) +0.101* Ln (AFP in ng/mL) +0.118*PT in second+0.050* Lg (HBV-DNA), cut-off values were 3.27 and 3.99.

### Model construction

The preoperative Very Early Recurrence Model of HCC (VERM-pre) was constructed based on the above eight risk factors and it was established through the AIC value test (11442.73). The nomogram is shown in Supplement Figure 2 (Supplemental Digital Content 3, http://links.lww.com/JS9/C392). The formula was established based on the β-estimate coefficient of each of these factors. Before conducting Cox regression, two conditions were required to be met: first, the proportional hazards assumption (PH assumption) needs to be satisfied; second, when the independent variable is a continuous variable, the relationship between the independent variable and the dependent variable in Cox regression should have a transformed linear relationship, which also needs to satisfy the following equation:


$$\;ln[h(t,X)/h_{0}\left(t\right)]=lnRR=\beta_{1}X_{1}+\beta_{2}X_{2}+\ldots +\beta_{m}X_{m}.$$

**Figure 1:**
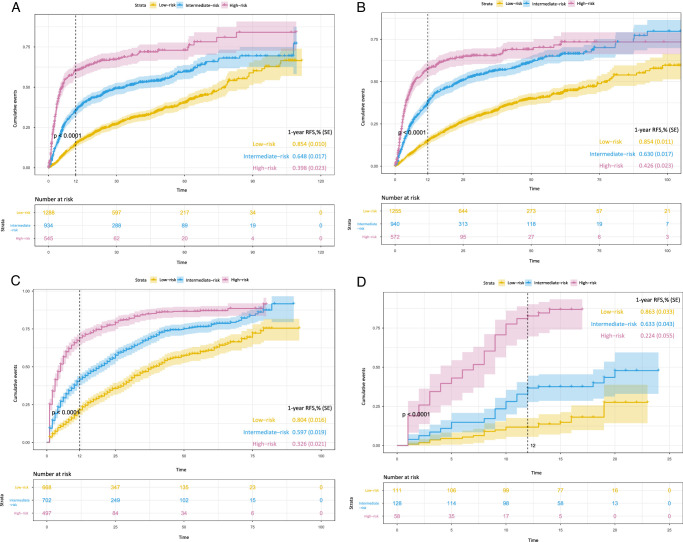
Kaplan–Meier plots of recurrence rate according to the three risk groups predicted by VERM-pre in different cohorts: (A) the training cohort; (B) the internal validation cohort; (C) the external validation cohort; (D) the prospective validation cohort.

In addition, logarithmic transformation can reduce the absolute values of the data for easier calculation and minimize the impact of large differential values on the equation. On the basis of these considerations, the 3 parameters (AFP, γ-GT, and HBV-DNA) were logarithmically transformed. And the transformation increased the performance of the model. The VERM-pre score was calculated using the formula and the optimal cut-off values were determined (3.27 and 3.99) through X-tile. Patients were then divided into the three risk groups (low-risk, intermediate-risk, and high-risk) by the cut-off values. The 1-year RFS was nearly 85% in the low-risk group, but only about 40% in the high-risk group (*P*<0.0001). These results are shown in Figure 1A–C. An automatic easy-to-use online calculator based on the VERM-pre score to provide the predictive recurrence rate at 1 to 12-month was then developed. It can generate a cumulative recurrence probability plot via the eight parameters and this online calculator is available on the website: https://dr-ray.shinyapps.io/VERM-pre/.

### Discrimination and calibration of VERM-pre

**Figure 2:**
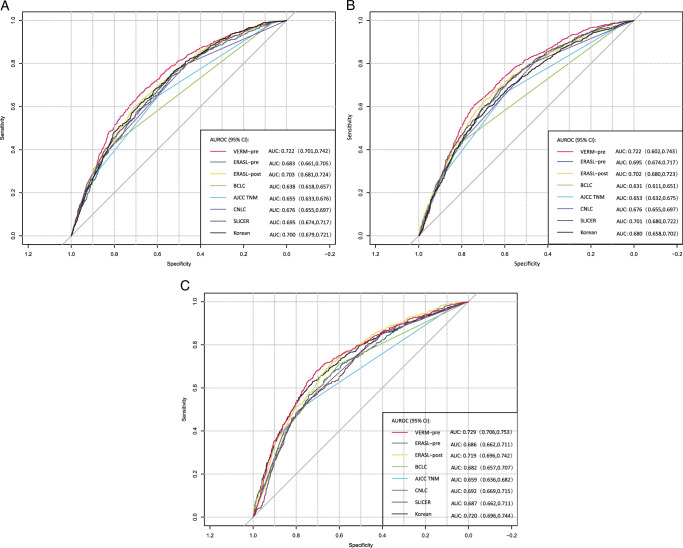
Comparation of AUROC at 12-month of VERM-pre with seven previous models in (A) the training cohort, (B) the internal validation cohort, and (C) the external validation cohort. AUROC, areas under time-dependent receiver operating characteristic curve.

**Figure 3:**
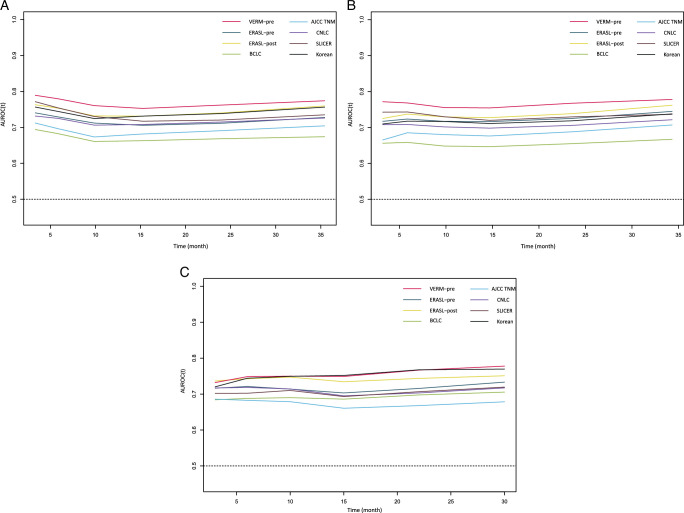
Time-dependent AUROC of the present score and seven previous models in (A) the training cohort, (B) the internal validation cohort, and (C) the external validation cohort. AUROC, areas under time-dependent receiver operating characteristic curve.

The VERM-pre model demonstrated good discriminative ability for both very early recurrence and OS, allowing patients to be classified into three distinct risk groups (Fig. 1 and Supplement Fig. 3 a–b, Supplemental Digital Content 4, http://links.lww.com/JS9/C393, http://links.lww.com/JS9/C394). The Harrell’s C-index and AUROC of the VERM-pre at different months are shown in Table 3 and Figs 2–3. The model was compared to the previously published models. The VERM-pre had higher C-indexes and AUROC values (0.722 and 0.722 at 12-month, Table 3 and Fig. 2). The time-dependent AUROC curve (Fig. 3) was also plotted to show the variations of the models’ discrimination ability. The capacity of discrimination of VERM-pre was relatively stable and was better than the seven other previously published models. The calibration plots are shown in Supplement Figure 4a–c (Supplemental Digital Content 5, http://links.lww.com/JS9/C395, http://links.lww.com/JS9/C396, http://links.lww.com/JS9/C397). The good agreements of the prediction probability of VERM-pre and the outcomes were observed in both the internal and external validation cohorts. As shown in DCA (Supplement Figures 5 a–c, Supplemental Digital Content 6, http://links.lww.com/JS9/C398, http://links.lww.com/JS9/C399, http://links.lww.com/JS9/C400), VERM-pre exhibited a positive net benefit in predicting 1-year postoperative recurrence, outperforming the seven previous models. This indicates its substantial clinical utility and practical value in the prediction of HCC recurrence.

**Table 3:** Comparation of performance of models.

		Time-dependent AUROC (95%CI)		
Models	C-index (95% CI)	t=3, m	t=6, m	t=12, m
VERM-pre				
Training	0.722 (0.630–0.813)	0.789 (0.764–0.815)	0.780 (0.757–0.802)	0.722 (0.701–0.742)
Internal validation	0.715 (0.625–0.805)	0.717 (0.747–0.796)	0.763 (0.747–0.790)	0.722 (0.602–0.743)
External validation	0.714 (0.696–0.732)	0.737 (0.706–0.768)	0.743 (0.718–0.768)	0.729 (0.706–0.753)
Prospective validation	0.771 (0.726–0.816)	0.811 (0.714–0.907)	0.803 (0.738–0.868)	0.831 (0.779–0.883)
ERASL-pre				
Training	0.682 (0.577–0.787)	0.741 (0.713–0.769)	0.729 (0.736–0.791)	0.683 (0.661–0.705)
Internal validation	0.680 (0.574–0.786)	0.717 (0.690–0.744)	0.723 (0.700–0.747)	0.695 (0.674–0.717)
External validation	0.670 (0.547–0.793)	0.717 (0.685–0.748)	0.721 (0.696–0.747)	0.686 (0.662–0.711)
ERASL-post				
Training	0.700 (0.600–0.800)	0.763 (0.736–0.791)	0.754 (0.730–0.777)	0.703 (0.681–0.724)
Internal validation	0.689 (0.588–0.790)	0.725 (0.697–0.753)	0.737 (0.714–0.761)	0.702 (0.680–0.723)
External validation	0.686 (0.575–0.797)	0.732 (0.703–0.762)	0.749 (0.724–0.773)	0.719 (0.696–0.742)
BCLC				
Training	0.638 (0.556–0.720)	0.694 (0.666–0.723)	0.683 (0.659–0.706)	0.638 (0.618–0.657)
Internal validation	0.675 (0.592–0.758)	0.656 (0.627–0.686)	0.659 (0.635–0.682)	0.631 (0.611–0.651)
External validation	0.652 (0.563–0.741)	0.684 (0.689–0.751)	0.688 (0.719–0.769)	0.682 (0.657–0.707)
AJCC TNM				
Training	0.652 (0.586–0.718)	0.712 (0.684–0.741)	0.696 (0.672–0.721)	0.655 (0.633–0.676)
Internal validation	0.645 (0.578–0.712)	0.665 (0.636–0.695)	0.685 (0.661–0.710)	0.653 (0.632–0.675)
External validation	0.697 (0.586–0.807)	0.686 (0.652–0.716)	0.683 (0.661–0.715)	0.659 (0.636–0.682)
CNLC				
Training	0.675 (0.628–0.722)	0.732 (0.704–0.760)	0.725 (0.701–0.749)	0.676 (0.655–0.697)
Internal validation	0.667 (0.620–0.714)	0.708 (0.680–0.735)	0.708 (0.685–0.732)	0.676 (0.655–0.697)
External validation	0.641 (0.590–0.692)	0.717 (0.655–0.717)	0.719 (0.657–0.708)	0.692 (0.669–0.715)
SLICER				
Training	0.698 (0.624–0.772)	0.772 (0.745–0.799)	0.754 (0.731–0.778)	0.695 (0.674–0.717)
Internal validation	0.692 (0.619–0.765)	0.742 (0.716–0.769)	0.743 (0.720–0.766)	0.701 (0.680–0.722)
External validation	0.669 (0.625–0.713)	0.702 (0.689–0.746)	0.702 (0.694–0.743)	0.687 (0.662–0.711)
Korean score				
Training	0.696 (0.588–0.804)	0.757 (0.729–0.784)	0.744 (0.721–0.768)	0.700 (0.679–0.721)
Internal validation	0.674 (0.567–0.781)	0.71 (0.681–0.739)	0.717 (0.693–0.742)	0.680 (0.658–0.702)
External validation	0.663 (0.660–0.666)	0.720 (0.671–0.733)	0.744 (0.668–0.718)	0.720 (0.696–0.744)

AJCC TNM, American Joint Committee on Cancer Tumor-Node-Metastasis; AUROC, areas under time-dependent receiver operating characteristic curve; BCLC, Barcelona Clinic Liver Cancer Staging; CNLC, Chinese Liver Cancer classification; ERASL, Early Recurrence After Surgery for Liver tumor; SLICER, Singapore Liver Cancer Recurrence; VERM, the Very Early Recurrence Model.

### Result of the prospective observational validation study

**Figure 4:**
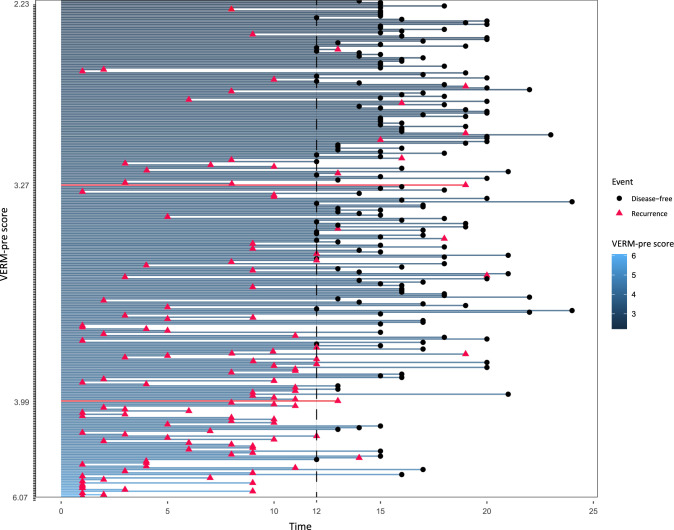
Swimming plot of the prospective validation cohort: the low-risk cohort VERM-pre score ≤3.27; the intermediate-risk cohort >3.27 and ≤3.99; the high-risk cohort >3.99. This big picture of the whole observational study showed that along with increasing of the VERM-pre score, the recurrence-risk elevated progressively.

In this study, we collected the limited variable parameters with a strong purpose (Table 1). Data of the eight independent factors were recorded and the VERM-pre score of each patient was calculated before the operation. Based on the model, patients were divided into three cohorts: 111 in the low-risk cohort, 128 in the intermediate-risk cohort, and 58 in the high-risk one (Fig. 1D). The KM plot (Fig. 1D) showed the three cohorts to have different cumulative events of cure and RFS rates and the outcomes were according with the prediction of VERM-pre. The C-index of VERM-pre in the prospective validation cohort was 0.771 (95% CI: 0.726–0.816) and the AUROC values at 3-month, 6-month, and 12-month were all above 0.8 (Table 3). The swimming plot (Fig. 4) illustrated a big picture of the whole observational study. Along with increasing of the VERM-pre score, the recurrence-risk elevated progressively. This further demonstrated the stable discrimination capacity of VERM-pre.

## Discussion

The Achilles’ heel of LR for HCC is the high incidence of postoperative tumor recurrence. The recurrence within 1-year after LR has been demonstrated to be an independent risk factor for worse OS by previous studies^8–10^. It is particularly unacceptable for patients who have just gone through the distressing experience and spending a lot of money for the surgery. Thus, it is important to come up with a well-validated preoperative prognostic model to predict the 1-year recurrence for clinicians to identify patients who have worse prognosis before surgery. In addition, this preoperative model can inform doctors and patients preoperatively on the possible recurrence rate so that additional interventions can be performed to prolong life expectancy by delaying tumor recurrence^30^.

The present study constructed a linear model using eight parameters which are commonly available before surgery. These parameters include tumor burden, imaging features, tumor biology and liver impairment status. Chan *et al*.^15^ and Shim *et al*.^13^ reported male as a prognostic risk factor. Huang *et al*.^29^ considered age as a risk factor and 42 years was the cutoff value. In our study, no demographic variables were found to be significant. Tumor burden as a risk factor is nearly included in every prognostic model. The Korean model incorporated tumor volume to reflect tumor burden and had tumor nodules as spherical in a logarithmically transformed formula^13^. The BCLC staging, AJCC TNM, CNLC, and ERASL scores, were similar to our model in using tumor diameter, tumor number, and intravascular invasion. A high level of AFP is known to be associated with poor prognosis and with tumor recurrence in HCC patients^8,31,32^. It reflects aggressive biological behavior of the tumor and it is associated with vascular invasion^33^. For construction of a scoring system, some researchers applied AFP as a categorical data, while SLICER stratified it into 4 grades (<10, ≤10 and <1000, ≤10000 and <10000, >1000), and SSCLIP into 2 grades (≤400 ng/ml, > 400 ng/ml)^14,29^. Similar to the ERSAL models, AFP was presented in our study as a continuous data with logarithmical transformation to construct the formula. In this way, the level of AFP crossed a larger range and had a lognormal distribution to improve the linear model fitting^16,17,34^. Liver function has always been evaluated by the Child-Pugh score and Albumin-Bilirubin (ALBI) score^15,28,29^. The latter score was found to have no significant association in our study. The prothrombin time has always been used as an indicator in defining liver dysfunction in the past^35^, and it has been utilized to construct the posthepatectomy liver failure predictive model^36^. It was also found to make a difference in our present model on long-term survival outcomes. γ-GT has rarely been used in predictive models. However, Lin *et al*.^37^ demonstrated that γ-GT levels above 41 U/l were relevant to an eightfold increased risk for HCC recurrence in a large scale of HBV cohort followed-up for 6 years. γ-GT levels reflect liver impairment and inflammatory-related status, a status which is involved in development of tumor via promoting angiogenesis and inhibiting apoptosis^30,38^. Liu *et al* ^39^. found a high level of γ-GT in predicting poor prognosis in HCC patients after hepatectomy when it was combined to be used with albumin and aspartate aminotransferase-to-lymphocyte ratio (ALRI). Similar to our results, Hu *et al*.^22^ demonstrated γ-GT was an independent risk factor for RFS. It was also reported as the substitutes for AFP after HCC treatment, especially with low AFP concentrations^40^. Satellite nodules are well-known recurrence predictors. Hu *et al*. reported satellite nodules as a risk factor as in the finding of our study. The identification of satellite nodules by radiology examinations in our study signified intrahepatic metastasis. This parameter implies the presence of other intrahepatic occult lesions which are difficult to confirm on radiology tests.

Many previously reported models were developed based on cohorts from one center which could lead to the homogeneous data problem. This issue would decrease the universal application of these models. A few studies were conducted based on multicenter analysis and used validated national-wide or world-wide cohorts. In such cases, it is important to stress the independent external validation in the calibration. In the past modeling analysis, there were some studies revalidating and recalibrating the former reported models retrospectively^9,10,17,41^. In our present study, the model was validated using cohorts from different centers and timing. To our knowledge, this is the first prospective validation study for the predictive prognostic model. VERM-pre was constructed and internally and externally validated retrospectively. The model had a better capacity of discrimination than the seven previously reported models, with higher C-indexes and AUROC values (Table 3). Furthermore, the analysis using DCA demonstrated that VERM-pre exhibits substantial predictive power. It was well-validated with large sample sizes. Through the scoring, patients were well divided into three recurrence-risk groups with significant differences among the groups. The high-risk group had only a RFS rate of 32–42%, while it was as high as 80–85% in the low-risk group. From this, a further prospective observational study was conducted. The swimming plot illustrated the follow-up outcomes of each patient which were basically consistent with the predictive score. Although this was a simple study, it improved the credibility and universal application of VERM-pre prospectively. The online calculator of VERM-pre also makes the dynamic prediction of recurrence easy to obtain.

There are limitations in this study. First, this study was initially designed as a retrospective study, which inherently could not avoid certain biases associated with such a design. Second, although the study has a relatively large sample size, it is still a single-country study, which cannot guarantee applicability of the findings to the Western world. Furthermore, the number of sample size used in the prospective validation was relatively small. This greatly affects its generalizability, and therefore, this model needs to be validated in the future by studies coming from other countries. Lastly, the present model was not compared with all the currently available prognostic models. For example, this model was not compared to the SSCIP score due to the lack of prothrombin time activity in many of our patients.

## Conclusions

The VERM-pre model predicted very early HCC recurrence in patients after ‘curative’ LR with a satisfactory capacity of discrimination. The model was validated using internal and external cohorts. The prospective observational validation study improved the model’s credibility and universal application. The online calculator makes it easy to use.

## Ethical approval

This study was approved by the Ethics Committee of Eastern Hepatobiliary Surgery Hospital (NO. EHBHKY 2023-K027- P001).

## Consent

Written informed consent was obtained from the patient for publication and any accompanying images. A copy of the written consent is available for review by the Editor-in- Chief of this journal on request.

## Sources of funding

This study was supported by the Science Fund for Creative Research Groups, NSFC, China, (81521091, 82073031); Clinical Research Plan of SHDC (No. SHDC2020CR5007, SHDC22020213). For the remaining authors none were declared.

## Author contribution

L.L.: data collection, data analysis or interpretation, and writing the paper; S.Q., K.L., Q.X.: provision of study materials, data collection, data analysis, or interpretation; Y.Y., J.C., Y.Z., S.Y.: provision of study materials, and writing – review and editing; B.X.: provision of study materials amd supervision; W.Y.L.: study concept or design, supervision, and writing – review and editing; W.Z.: study concept or design, project administration, supervision, and writing – review and editing.

## Conflict of interest statement

There is nothing to disclose. No conflict of interest exits in the submission of this manuscript, and this manuscript has been approved by all authors for publication.

## Research registration unique identifying number (UIN)

This study was registered in the Chinese Trial Registry from http:// www.chictr.org.cn (ChiCTR2300077747) and conducted in line with the STROCSS 2021 criteria.

## Guarantor

Weiping Zhou.

## Data availability statement

Authors confirm that any datasets generated during and/or analyzed during the current study are available upon reasonable request.

## Provenance and peer review

None.

## Category

The manuscript is being submitted as an original article.

## Statement

The paper is not based on any previous communication to a society or meeting.

## Supplementary Material
