# Supplementary figures and images for: Development and comprehensive validation of a predictive prognosis model for very early HCC recurrence within one year after curative resection: a multicenter cohort study
Source: Int J Surg. 2024 Apr 15;110(6):3401–11. doi: 10.1097/JS9.0000000000001467 (PMC11175792; doi:10.1097/JS9.0000000000001467)

A

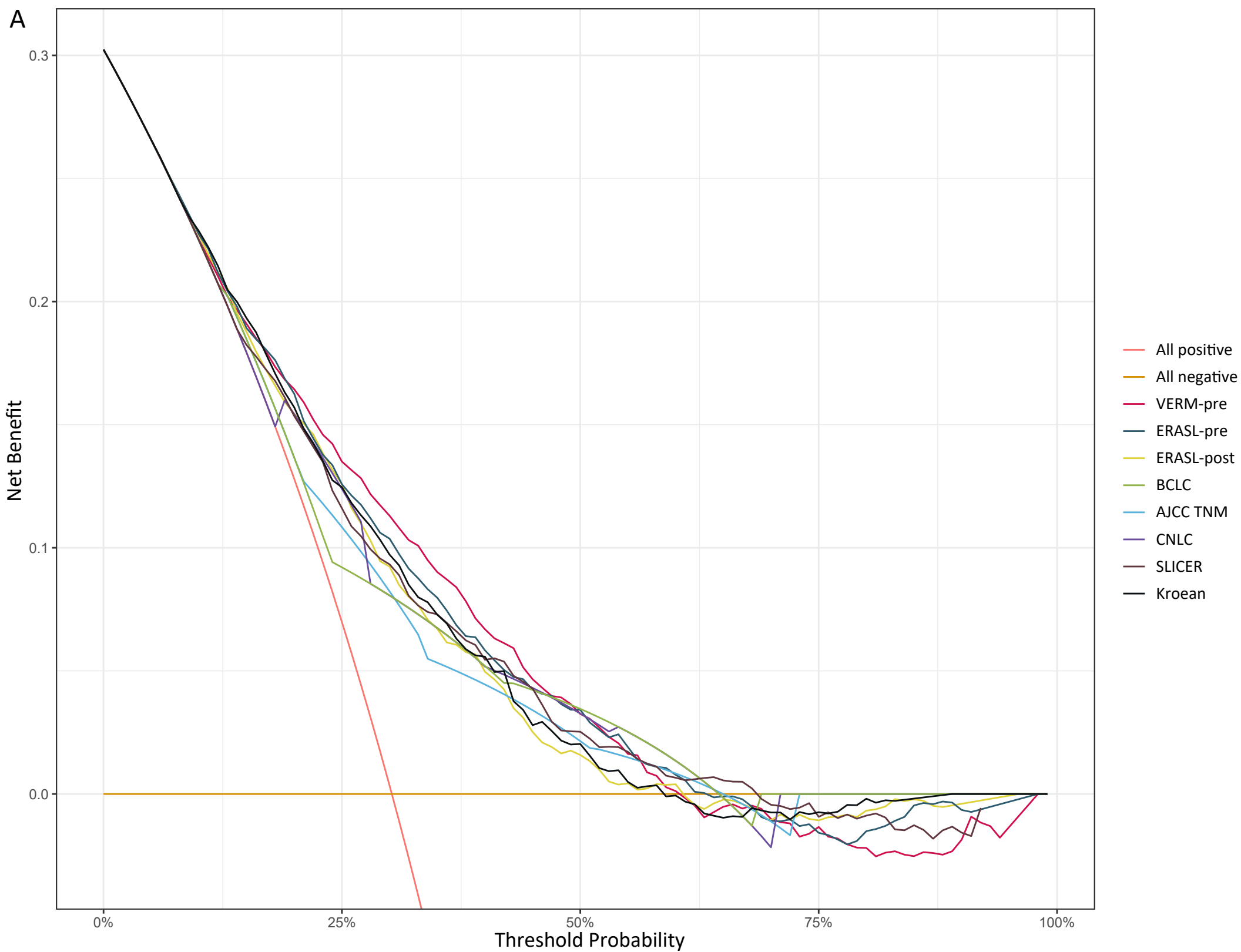

Supplement: Supplementary file 8 [file js9-110-3401-s008.pdf]
