# Supplementary material for: Development and comprehensive validation of a predictive prognosis model for very early HCC recurrence within one year after curative resection: a multicenter cohort study
Source: Int J Surg. 2024 Apr 15;110(6):3401–11. doi: 10.1097/JS9.0000000000001467 (PMC11175792; doi:10.1097/JS9.0000000000001467)
Supplement: Supplementary file 11 [file js9-110-3401-s011.docx]

| Supplement table 1. Univariable analysis of prognostic parameters for the model in the training cohort | | | | |
| --- | --- | --- | --- | --- |
| Parameters |  | Univariable analysis | | |
|  |  | Hazard Ratio (95% CI) | Coefficient (95% CI) | P value |
| Age |  | 0.995(0.988, 1.002) | -0.016(-0.022, 0.010) | 0.134 |
| Gender | male | 1.168(0.934,1.460) | 0.155(-0.068,0.378) | 0.174 |
| Hepatitis B | positive | 1.036 (0.841,1.276) | 0.035(-0.172,0.242) | 0.740 |
| Hepatitis C | positive | 1.407(0.882,2.246) | 0.342(-0.126,0.809) | 0.152 |
| Macrovascular invasion | Yes | 2.045(1.687,2.478) | 0.715(0.523,0.907) | **<0.001** |
| Tumor number |  | 1.130(1.042,1.226) | 0.122(0.040,0.204) | **0.003** |
| Tumor diameter |  | 1.064(1.044,1.084) | 0.062(0.042,0.082) | **<0.001** |
| Tumor capsule | uncomplete | 1.147(1.014,1.296) | 0.137(0.014,0.260) | **0.060** |
| Satellite nodule | Yes | 1.168(0.991,1.375) | 0.155(-0.010,0.320) | **0.030** |
| Platelet |  | 1.001(0.999,1.002) | 0.001(0.000,0.002) | 0.230 |
| Total bilirubin |  | 0.999(0.994,1.004) | 0.001(-0.005,0.007) | 0.771 |
| Albumin |  | 1.000(0.979,1.022) | -0.0001(-0.020,0.020) | 0.997 |
| Ln (γ-GT) |  | 1.172(1.066,1.289) | 0.159(0.063,0.255) | **0.001** |
| Ln (AFP) |  | 1.104(1.067,1.142) | 0.099(0.066,0.132) | **<0.001** |
| PT |  | 1.126(1.045,1.213) | 0.119(0.045,0.193) | **0.002** |
| Lg (HBV-DNA) |  | 1.048(0.999,1.099) | 0.047(0.00004,0.094) | **0.052** |
| γ-GT, γ-glutamyl transpeptidase; AFP, alpha-fetoprotein; PT, prothrombin time; HBV-DNA, hepatitis B virus deoxyribonucleic acid. | | | | |
|  |  |  |  |  |
